# Secretory factors released from high dose radiation-activated osteoclasts increase the expression level of pain-associated neuropeptides in sensory neuronal cultures

**DOI:** 10.21203/rs.3.rs-4534694/v1

**Published:** 2024-07-01

**Authors:** Sun H Park, Megan Peters, Caleb Aguayo, Michael K Farris, Ryan T Hughes, Joseph Moore, Michael T Munley, Kaitlyn E Reno, Jean Gardin, J Mark Cline, Christopher M Peters, Jeffrey S Willey

**Affiliations:** Wake Forest University School of Medicine

**Keywords:** Stereotactic Body Radiation Therapy, chest wall pain, osteoclasts, sensory neurons, bisphosphonates, neuropeptides

## Abstract

Stereotactic Body Radiation Therapy for lung tumors near the chest wall often causes significant chest wall pain (CWP), negatively impacting patients’ quality of life. The mechanisms behind SBRT-induced CWP remain unclear and may involve multiple factors. We investigated the potential crosstalk between radiation-activated osteoclasts and sensory neurons, focusing on osteoclast-derived factors in CWP. Using the murine pre-osteoclast cell line Raw264.7, we induced differentiation with RANKL, followed by 10Gy gamma-irradiation. Conditioned media from these irradiated osteoclasts was used to treat sensory neuronal cultures from mouse dorsal root ganglia. Neuronal cultures were also directly exposed to 10Gy radiation, with and without osteoclast co-culture. Analysis of osteoclast markers and pain-associated neuropeptides was conducted using RT-qPCR and histochemical staining. Osteoclast differentiation and activity were inhibited using Osteoprotegerin and risedronate. Results showed that high-dose radiation significantly increased osteoclast size, resorption pit size, and activity biomarkers. Neurons treated with CM from irradiated osteoclasts showed increased expression of pain-associated neuropeptides CGRP and Substance P, which was mitigated by osteoprotegerin and risedronate. This study suggests that high-dose radiation enhances osteoclast activity, upregulating pain-associated neuropeptides in sensory neurons, and that inhibitors like osteoprotegerin and risedronate may offer therapeutic strategies for managing radiation-induced pain.

## INTRODUCTION

Stereotactic Body Radiation Therapy (SBRT) has become a standard treatment option for patients with lung tumors who decline or are ineligible for surgical resection ^1^. For peripheral lung tumors in close proximity to the chest wall, SBRT often unavoidably delivers high doses of radiation to the normal ribs or vertebrae near the targeted tumor. This can predispose to significant chest wall pain (CWP), radiation induced rib fractures (RIRF) or even vertebral fractures ^2–4^.

Rates of chest wall toxicity have been correlated with multiple chest wall dosimetric parameters. Approximately one third of patients develop clinically-relevant CWP when greater than 30 cm3 of chest wall receives 30 Gy or more ^4^. In routine practice, it is common to prioritize comprehensive target coverage over sparing of non-critical organs at risk such as the chest wall and ribs in order to maintain optimal tumor control outcomes ^5,6^. However some clinicians may consider lowering their total RT dose or using less effective protracted fractionation schemes to avoid severe chest wall toxicity, which has been associated with higher risks of poor survival outcomes ^5^. CWP usually manifest between 6–9 months post-treatment, and may occur with or without evidence of rib fractures ^4,7–10^. The incidence of RIRF within two years after SBRT is as high as 40% ^11^. CWP associated with RIRF presents as acute onset of severe, sharp pain that often persists until the fractures heal. SBRT-associated RIRFs demonstrate delayed healing in comparison to traumatic fractures; only 47% of RIRF had healed during a median 49-month follow-up period in one study ^2^. On the other hand, CWP without obvious radiologic signs of rib fracture may present with acute, focal sharp pain over a few days to weeks, or alternatively as chronic, dull pain for years. Both manifestations of CWP have the potential to impact patients’ quality of life ^4,11,12^. Severe pain may lead to compromised ventilation, decreased oxygen saturation, as well as risks of atelectasis and infection ^13^. Pain management can be challenging and may involve non-steroidal anti-inflammatory medications, systemic corticosteroids, gabapentin, narcotics, and/or percutaneous nerve blocks ^11,14^. Importantly, these treatments only offer symptomatic relief without addressing the root cause of the CWP. This underscores the urgent need for alternative targeted strategies to mitigate the risk of SBRT-related CWP/RIRF without compromising cancer control.

The exact origins of SBRT-induced CWP are not fully understood, though some hypotheses include: acute localized inflammation, dysregulated bone turnover through differential effects on osteoclasts and osteoblasts, fibrotic changes in subcutaneous soft tissues or nerve fibers, or a complex mix of all of these mechanisms simultaneously ^11,15–17^. Our prior research indicates that low-dose radiation exposure can lead to an increase in osteoclast number and activity, resulting in increased bone degradation ^18–23^. Studies have found that the preemptive administration of bisphosphonates can counter radiation-induced bone loss in animal models ^24^. This suggests that early interventions targeting osteoclast activation might help prevent CWP, especially those cases associated with rib fractures, and this has been an area of active clinical research ^25–27^. However, this does not account for CWP cases that manifest without fractures. Active osteoclasts may release factors promoting sensory nerve growth and pain. For example, exosomes from osteoclasts have been implicated in nerve growth and inflammatory pain in animal models of osteoarthritis ^28^. Furthermore, the acidosis caused by protons released from active osteoclasts has links with both cancer-induced bone pain ^29^ and osteoclast derived molecules induces nerve sprouting and pain associated with osteoarthritis ^30^. The potential role of radiation-activated osteoclasts in CWP remains unexplored. Our hypothesis is that high-dose radiation might enhance osteoclast activity, causing them to release secretory factors that activate pain signaling in sensory neurons.

## RESULTS

### Impact of High-Dose Radiation on Osteoclast Maturation and Activity

**Figure 1:**
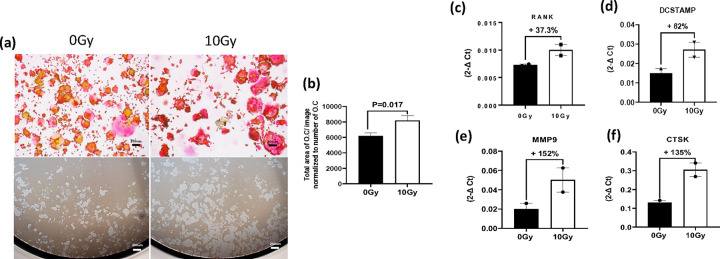
Maturation and activation of osteoclasts following high-dose radiation exposure. (**a**) Representative images showing TRAP staining (top panel) and resorption pit data (bottom panel) for osteoclasts 48 hours after exposure to either no radiation (0 Gy) or a single fraction of 10 Gy radiation. Scale bar = 200μm (**b**) Quantification of the total area of TRAP-positive mature osteoclasts exposed to no radiation therapy (NRT) or radiation therapy (RT). (**c-f**) RT-qPCR analysis of osteoclasts exposed to NRT or RT, illustrating changes in the expression of genes associated with osteoclast differentiation and maturation (RANK and DCstamp), as well as activity (MMP9, CTSK). The experiment was conducted two times in triplicate. Levels of statistical significance are indicated as *P ≤ 0.05.

To assess the impact of high-dose radiation on osteoclast activity, pre-osteoclast Raw264.7 cells were cultured with 35ng/ml of RANKL. Following three days of culturing, cells were subjected to radiation as described in method. Forty-eight hours post-irradiation, cells were stained with TRAP staining solution to identify mature osteoclasts. We observed a significant increase in the size of TRAP-positive osteoclasts in irradiated cells compared to controls (Fig. 1A and B). Furthermore, when irradiated cells were plated on bone biomimetic synthetic surfaces, a notable augmentation in resorption pit size was evident compared to untreated control cells (Fig. 1A). Complementing these morphological observations, RT-qPCR analysis revealed a robust upregulation in the expression of osteoclast biomarkers associated with differentiation (RANK), fusion (DcStamp), and resorption activity (CTSK and MMP9) in irradiated cells compared to control cells (Fig. 1C–F).

### Impact of Radiation-Activated Osteoclast-Derived Secretory Factors on Pain-Associated Neuropeptides in Sensory Neurons

Building upon previous studies indicating the involvement of secretory factors released from activated osteoclasts in modulating sensory neurons under various pathological conditions, we sought to investigate the impact of radiation-activated osteoclast-derived secretory factors on pain-associated parameters. CM from osteoclasts exposed to 10 Gy radiation were collected and utilized to treat primary sensory neuronal cultures derived from DRGs. Control cultures received CM from non-irradiated osteoclasts. Subsequently, DRG neuronal cultures were fixed and stained with neuronal markers. β-III tubulin served as a pan-neuronal marker, CGRP, a marker of peptidergic unmyelinated C-fibers and thinly myelinated A-fibers, and NF200 identified myelinated A-fibers. Also, we specifically measured the levels of pain associated molecules including neuropeptides (CGRP and SP) and nerve growth factor (NGF) as they are well-documented to play crucial roles in mediating pain transmission and modulation. Elevated expression of these neuropeptides has been linked to heightened pain sensitivity in various pain states, hence their assessment served as a potential indicator of pain-associated responses elicited by radiation-activated osteoclast-derived secretory factors.

**Figure 2:**
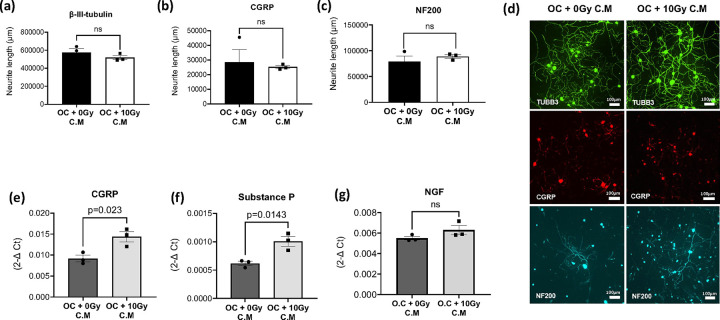
Impact of radiation-activated osteoclasts on sensory neuronal cultures. Immunofluorescence analysis of mouse DRG-derived sensory neuronal cultures exposed to conditioned media (C.M) from osteoclasts treated with either no radiation (0 Gy) or 10 Gy radiation. After 48 hours, cells were stained with antibodies against neuronal markers: (**a**) β-III tubulin, (**b**) CGRP, and (**c**) NF200, followed by microscopic image analysis. (**d**) Representative images of sensory neuronal cultures stained with these neuronal markers, β-III tubulin (TUBB3), CGRP, and NF200. The experiment was conducted two times in triplicate. (**e-g**) mRNA expression levels of pain-associated molecules (CGRP, SP, and NGF) in neuronal cultures exposed to C.M from radiation-activated osteoclasts. The experiment was conducted three times in triplicate. Levels of statistical significance are indicated as *P ≤ 0.05.

Conditioned media was examined to determine if secretory factors derived from radiation-activated osteoclasts may contribute to pain responses by upregulating the expression of pain-associated neuropeptides in sensory neurons. CM derived from radiation-activated osteoclasts did not exert a significant impact on the growth of sensory neurons (Fig. 2A–D), as evidenced by no change in total neurite length. However, it notably increased the mRNA expression of CGRP and SP (Fig. 2E and F). No significant change in the expression level of nerve growth factor (NGF) was observed (Fig. 2G).

### Modulation of Radiation-Induced Osteoclast Activation and Neuronal Responses by OPG and Bisphosphonate

**Figure 3:**
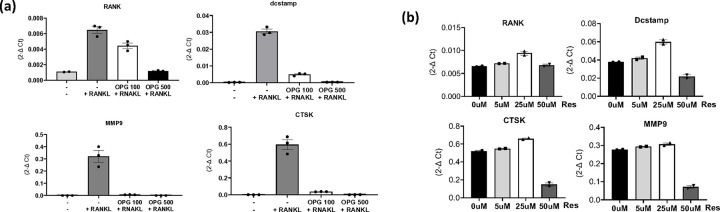
Optimization of OPG and risedronate concentrations on RANKL-induced osteoclast differentiation and activity. (**a**) mRNA expression levels of mouse osteoclast-associated biomarkers influenced by varying concentrations of OPG. The experiment was conducted one time in triplicate. (**b**) mRNA expression levels influenced by different concentrations of risedronate (Res). The experiment was conducted one time in duplicate.

To determine if activated osteoclasts (OC) upregulate neuropeptides in neuronal cultures and test the potential of OPG and risedronate as modulators of radiation-induced osteoclast activation and subsequent neuronal responses, OCs were pre-treated with either OPG or risedronate before radiation exposure. First, the concentrations of OPG and risedronate required to inhibit differentiation and activity of osteoclast induced by RANKL were determined. Pre-treatment with 500μM OPG effectively prevented both RANKL-mediated osteoclast differentiation and activity, as evidenced by decreased expression of differentiation and activity markers (Fig. 3A). Conversely, risedronate, up to a concentration of 50μM, did not impact osteoclast differentiation but effectively inhibited RANKL-mediated osteoclast activity, indicated by decreased expression of MMP9 and CTSK (Fig. 3B). Subsequently, the effects of OPG and risedronate on radiation-induced osteoclast differentiation, maturation, and activity were investigated.

**Figure 4:**
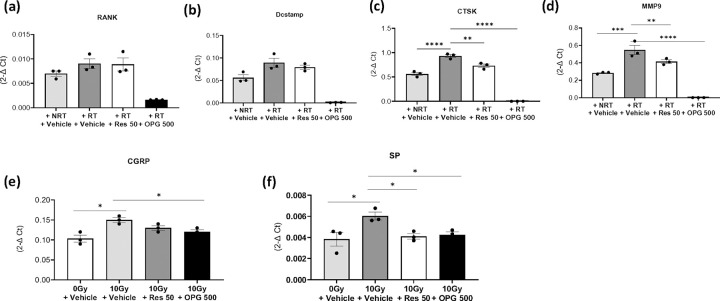
Reduction in neuropeptide expression by osteoclast inhibitors in radiation-activated osteoclast-derived CM. (**a-d**) RT-qPCR analysis of osteoclasts activated by 10 Gy radiation and pre-treated with either OPG (500 μg/ml) or risedronate (50 μM). The experiment was conducted two times in triplicate. (**e and f**) mRNA expression levels of CGRP and Substance P in murine neuronal cultures exposed to CM from osteoclasts treated with either no radiation or 10 Gy radiation and pre-treated with OPG or risedronate. The experiment with OPG and risedronate was conducted one time in triplicate. Levels of statistical significance are indicated as *P≤0.05, **P≤0.005, ***P≤0.0001.

Pre-treatment with OPG (500uM) significantly attenuated the upregulation of differentiation and activity markers observed in irradiated osteoclasts (Fig. 4A–D). Similarly, pre-treatment with risedronate decreased the radiation-induced expression of osteoclast activity markers (CTSK, MMP9) without significantly impacting differentiation/maturation markers (RANK and DCstamp) (Fig. 4A–D).

Of particular interest, when neuronal cultures were exposed to CM derived from irradiated osteoclasts pre-treated with risedronate or OPG, a notable reduction in the expression of CGRP and SP was observed, resembling the levels seen in neuronal cultures treated with control CM (Fig. 4E and F).

### Effect of Direct Radiation Exposure on Sensory Neurons and Neuropeptide Expression

Previous investigations primarily focused on elucidating the impact of activated osteoclasts on neuronal cultures. However, considering the clinical context where both ribs and nerves are concurrently exposed to radiation near targeted tumors, it becomes imperative to understand the direct effects of radiation on neurons and its potential role in chest wall pain post-radiation.

**Figure 5:**
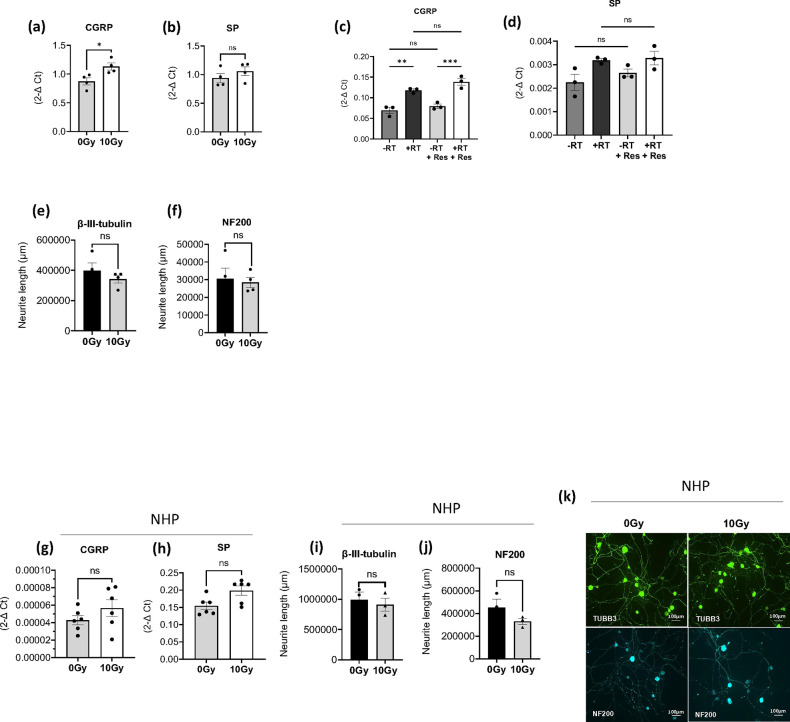
Direct effects of radiation on neuropeptide expression in sensory neurons. (**a-b**) mRNA expression levels of CGRP and SP in murine sensory neuronal cultures directly exposed to 10 Gy radiation compared to unexposed cells (0 Gy). The experiment was conducted two times in quadruplicate. (**c-d**) Gene expression levels of CGRP and SP in sensory neurons following radiation and pre-treatment with risedronate (50 μM). The experiment was conducted two times in duplicate. (**e-f**) Analysis of neurite length post-radiation in murine sensory neuronal cultures stained with antibodies against β-III tubulin and NF200 by immunofluorescence. The experiment was conducted two times in quadruplicate. (**g-h**) mRNA expression levels of CGRP and SP in non-human primate sensory neuronal cultures directly exposed to 10 Gy radiation compared to unexposed cells (0 Gy). The experiment was conducted one time in six replicates. (**i-j**) Neurite length analysis post-radiation in non-human primate (NHP) sensory neuronal cultures. (**k**) Representative images of NHP sensory neuronal cultures stained with these neuronal markers, β-III tubulin (TUBB3) and NF200. Levels of statistical significance are indicated as *P≤0.05. The experiment was conducted one time in triplicate.

To address these questions, sensory neuronal cultures were exposed to 10 Gy radiation, and subsequent characterization was conducted to assess nerve growth and gene expression changes. Analysis performed 48 hours post-radiation through RT-qPCR revealed that direct radiation exposure led to an increase in the mRNA expression of neuropeptides CGRP and SP, with particularly notable elevation in CGRP levels reaching statistical significance (Fig. 5A and B). As expected, risedronate pre-treatment exhibited no discernible effect on neuropeptide expression (Fig. 5C and D). In addition, immunofluorescence studies indicated that change in total neurite length of sensory neurons stained with β-III tubulin and NF200 post-radiation was not statistically significant (Fig. 5E and F). Similar observations were made in neuronal cultures derived from two different non-human primates exposed to the same radiation dose, though none of the changes reached statistical significance (Fig. 5G–K).

### Effect of Radiation Exposure on Osteoclast-Sensory Neuron Co-Cultures

To replicate the clinical scenario of simultaneous radiation exposure of ribs and intercostal nerves during SBRT to a peripheral lung tumor, osteoclasts and sensory neurons were co-cultured without direct contact. Following simultaneous radiation treatment (10 Gy) of both cell types, media exchange was performed to examine the effect of secretory factors from activated osteoclasts on sensory neurons. After 48 hours of media sharing, the expression levels of neuropeptides in neuronal cultures were assessed.

**Figure 6:**
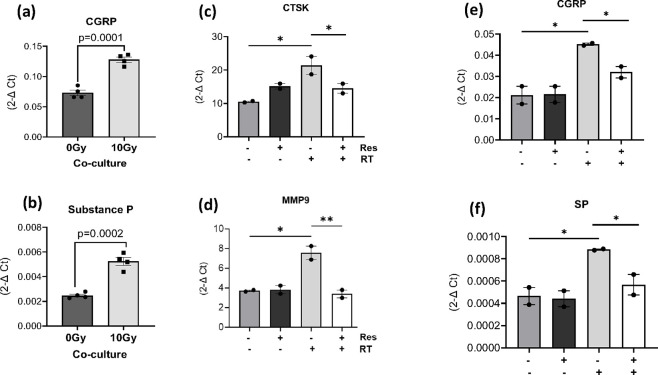
Inhibition of neuropeptide expression in sensory neurons co-cultured with osteoclasts by risedronate. (**a-b**) Gene expression levels of CGRP and SP in murine sensory neurons co-cultured with osteoclasts following simultaneous radiation treatment. The experiment was conducted two times in quadruplicate. Gene expression levels of (**c-d**) osteoclast activity markers (MMP9 and CTSK) in osteoclasts, and (**e-f**) pain-associated neuropeptides, CGRP and SP in neurons from OC-neuron co-cultures following simultaneous radiation treatment and pre-treatment with risedronate (50 μM). Levels of statistical significance are indicated as *P≤0.05, **P≤0.005. The experiment with risedronate was performed one time in duplicate.

Consistent with previous results utilizing conditioned media (Fig. 2E and F), a significant increase in the gene expression levels of CGRP and SP was observed when cells were irradiated compared to the control without radiation treatment (Fig. 6A and B). Additionally, when co-cultures were treated with risedronate, a marked inhibition of radiation-induced osteoclast activity was evident, as indicated by decreased expression levels of CTSK and MMP9 (Fig. 6C and D). Interestingly, the presence of risedronate in co-cultures also significantly mitigated the radiation-induced increase in expression of CGRP and SP, similar level to the non-irradiated cells (Fig. 6E and F). These findings collectively suggest that bisphosphonate intervention can effectively counteract the radiation-induced increase in pain-associated neuropeptides, presumably by inhibiting osteoclast activity. These findings further suggest that the reduction in neuropeptide expression by risedronate in co-cultures post-radiation exposure predominantly arises from its inhibitory effect on osteoclasts.

### Impact of Osteoclast-Derived Secretory Factors on Irradiated Neurons

**Figure 7:**
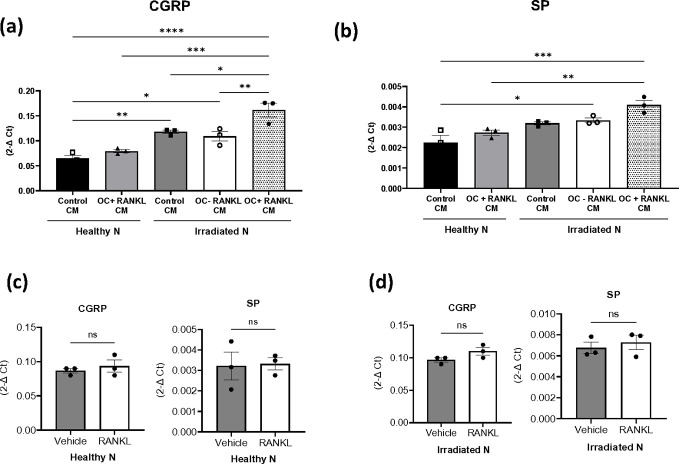
Enhanced neuropeptide expression by osteoclast-derived factors in irradiated neurons. **(a-b)** Expression levels of CGRP and SP in both non-irradiated and irradiated murine sensory neurons treated with CM from RANKL-differentiated osteoclasts. (**c-d**) Impact of RANKL alone on neuropeptide mRNA expression in healthy and irradiated sensory neurons. Levels of statistical significance are indicated as *P≤0.05, **P≤0.005. The experiment was conducted two times in triplicate.

Recognizing that damaged neurons are more sensitive to surrounding factors due to altered signaling pathways, increased excitability, and impaired repair mechanisms, we hypothesized that irradiated neurons may respond differently to factors secreted by osteoclasts than non-irradiated neurons. Understanding the complexity of interactions between these two cell types in the clinical setting after SBRT, where tissues near the treatment site may interact with those farther away, prompted us to explore the impact of secretory factors from adjacent bone cells on damaged nerves. Irradiated and non-irradiated sensory neuronal cultures were exposed to conditioned media from mature osteoclasts (non-irradiated). We observed that sensory neurons subjected to radiation exhibited significantly higher expression of both CGRP and SP after exposure to CM derived from RANKL-induced mature osteoclasts compared to healthy neurons (Fig. 7A and B). To ascertain whether the increase in peptide expression induced by RANKL-induced osteoclast CM was solely attributed to RANKL, we treated both healthy and irradiated neurons with RANKL alone. However, no change in peptide expression was observed (Fig. 7C and D).

## DISCUSSION

Our study explores the relationship between osteoclasts and sensory neurons after exposure to high dose radiation, and we aimed to identify potential mechanisms that underlie the development of CWP following SBRT to tumors near the chest wall. First, we demonstrated that high dose radiation stimulated osteoclast maturation and activity in agreement with our prior low dose per fraction rodent models ^24,31^. The resulting dysregulation in bone turnover compromises structural integrity and likely is the primary cause of SBRT-induced rib and vertebral fractures. While RIRF manifests as acute, severe CWP that often takes months to resolve, patients commonly report both acute and chronic forms of CWP in the absence of any radiologically apparent fractures ^4,32^. Potential etiologies of post-SBRT CWP without clear fracture include acute localized inflammation, occult microfractures, or direct damage and fibrosis of subcutaneous soft tissues and intercostal nerves. Routine therapeutic interventions include escalating trials of anti-inflammatory medications (non-steroidal anti-inflammatory medications, or corticol-steroids), and narcotics (ARRO). Usually, these are effective strategies and pain is self-limited over weeks to months, but may lead to unwanted side effects including gastric ulcers, renal/liver toxicity, immunosuppression, and in the case of opioid use disorder ^33^. In the setting of chronic refractory post SBRT pain, intercostal nerve block, or gabapentin have been utilized, the efficacy of the latter suggesting a neuropathic mechanism ^8,34^. Welsh *et al* described a correlation between pre-SBRT obesity and diabetes with higher rates of chronic CWP development, suggesting that underlying subclinical or clinical neuropathy may lower the threshold for radiation-induced nerve damage to contribute to CWP.

Prior studies outside of the context of bone irradiation have validated that upregulated osteoclast activity from other pathologies such as osteoporosis and osteoarthritis can modulate the expression of CGRP, SP, and/or NGF, contributing to pain sensitivity ^35–37^. Additionally, increased abnormal nerve sprouting has been linked to osteoclast activity ^28^. Together, these findings suggest that osteoclasts contribute to pain in more complex ways than just loss of structural integrity through resorption.

The present study is unique in demonstrating substantial cross-talk between upregulated osteoclasts and irradiated and unirradiated nerves. We demonstrated that direct irradiation to neurons also alters the levels of pain associated markers and nerve growth, suggesting that nerves directly damaged by radiation are more sensitive to secretory factors from mature osteoclasts which leads to higher levels of expression of pain associated markers such as CGRP and SP as shown in Fig. 7A and B.

Our clinical data seems to support this osteoclast neuron cross talk mechanism as well. In a randomized double-blind clinical trial at our institution, 76 patients receiving SBRT for peripheral lung tumors were treated with placebo or risedronate (150 mg single dose 2 weeks prior to SBRT). While there was no difference in the rate of RIRF (overall 34%), risedronate was associated with a significant reduction in modified CTCAE grade 2 or higher CWP (42 vs 18%, p = 0.045) (Unpublished abstract). As the first study to evaluate the use of bisphosphonates to reduce the risk of radiation induced bone loss after peripheral lung SBRT, a standard dosing or regimen for the bone-protecting agent was not available at the time of trail design, and it is possible that the dose of risedronate was inadequate, which may explain the similar RIRF rates. If RT-mediated activation of osteoclasts does in fact contribute to both RIRF and at least some component of CWP without fractures, this presents osteoclast inhibition as an attractive potential therapeutic target for patients with tumors adjacent to bone and nerve.

Our current study findings emphasize the therapeutic potential of osteoclast inhibitors using bisphosphonates in managing CWP as well. Risedronate, which inhibits osteoclast activity significantly decreased the expression of neuropeptides induced by osteoclast-derived factors in neurons. Although risedronate did not directly affect the CGRP and SP levels increased after radiation exposure in neurons, it significantly inhibited CGRP and SP in sensory neurons in co-culture with osteoclasts after radiation treatment. The extent to which direct radiation exposure and the neuropeptide and nerve growth changes contribute to CWP is unclear and warrants further study. The neuropeptides increase induced by osteoclast-derived factors and/or direct irradiation might lead to different functional outcomes. It would be important to better understand the component of CWP caused by direct nerve damage after SBRT versus an alternative pathway in which neuropeptides released from neurons in close proximity to activated osteoclasts act on pain signaling.

Currently we are pursuing animal models of high-dose exposure to the chest wall concurrent with various doses/schedules of bisphosphonates to identify a prophylactic effect on CWP or RIRF and elucidate an appropriate agent and dose for future clinical study. A better understanding of the agent, dose, and timing most suitable for future study would inform the design of a larger trial specifically powered for reduction in CWP/RIRF for patients with tumors immediately adjacent to the chest wall planned for SBRT. If successful, this would be a valuable and immediately implementable strategy for clinicians to reduce the risk of chest wall toxicity after SBRT while maintaining excellent cancer control rates, particularly given the well-established use and safety profile of bisphosphonate therapy in other diseases.

In conclusion, our study presents unique evidence that radiation-induced osteoclast activation continues to neuronal cross-talk with neurons that may contribute to CWP after lung SBRT near the chest wall, particularly in patients without evidence of fracture. Osteoclast inhibition may represent a novel strategy not only to reduce radiation-induced bone loss and fracture, but potentially prevent or relieve CWP through neuromodulation of osteoclast-derived, radiation induced pain signaling.

## MATERIALS AND METHODS

All animal work was performed as approved by the Wake Forest University School of Medicine Institutional Animal Care and Use Committee (IACUC). The study was carried out in compliance with the ARRIVE guidelines and in accordance with relevant guidelines and regulations.

### Sensory neuronal cultures from mice dorsal root ganglia (DRG)

Mice were purchased from Jackson laboratories (Bar Harbor, ME, USA). Mice were euthanized with CO_2_ followed by cervical dislocation, in accordance with IACUC guidelines. Primary DRG neuronal culture was prepared following the previously published protocol with modifications ^38^. Thoracic DRG (T1-T13) of male C57BL/6 mice (8–12 weeks old) were dissected followed by enzymatic digestions: 1] tissues were incubated in 3 mL of papain solution containing 30 U/mL papain (Worthington Biochemical Corp., Lakewood, NJ), 0.1% saturated NaHCO3 solution (Sigma-Aldrich, St. Louis, MO), and 0.3 mg/mL L-Cys (Sigma-Aldrich) for 30 min at 37°C with 5% CO2, and 2] incubated in 3 mL of collagenase type II (CLS2)/dispase type II (Dispase II) solution containing 4 mg/mL CLS2 (Worthington Biochemical Corp.) and 4.7 mg/mL dispase (Sigma-Aldrich) for 30 min at 37°C with 5% CO2. DRG tissues were triturated and filtered through a stainless mesh sieve (40 μm, Thermo Fisher Scientific) to obtain single-cell suspensions. Cells incubated with myelin beads (Miltenyl tech) in BSA buffer for 10mins @ 4C following the company’s protocol. The bead/cell suspension went through the magnetic column to remove myelin debris and the eluted cell suspension was washed and counted. 2000–5,000 cells of DRGs in 30 μL of warm neuronal growth (NG) medium [Neurobasal-A (Thermo Fisher Scientific, Gibco), 1% N2 (Thermo Fisher Scientific), 2% B-27 (Thermo Fisher Scientific), 2 mM L-glutamine (Thermo Fisher Scientific), 1% penicillin-streptomycin (Thermo Fisher Scientific), and 0.4% glucose (Sigma-Aldrich)] were seeded onto the center of 12 mm round coverslips (MatTek Corp., Ashland, MA), pre-coated with Poly-D-lysine (50 ug/mL, overnight at 4°C, Thermo Fisher Scientific, Corning) and laminin (20 μg/mL, > 1 h at room temperature, Thermo Fisher Scientific, Corning), in 24-well plate. After 1–2 h, 1 mL of warm NG medium was gently added to the sides of wells and the cells were maintained at 37°C with 5% CO2. 48hr after cells were seeded, cells were either treated with radiation using Cs137 γ-rays and/or conditioned media of radiation activated osteoclasts for further studies. For co-cultures, 2000 cells/60ul growth media were plated on μ-Slide 2 Well Co-Culture plates (81806, iBidi USA, Inc. Fitchburg, WI).

### Sensory neuronal cultures from non-human primate (NHP) DRGs

Dorsal root ganglia were harvested from rhesus macaques (Macaca mulatta) non-human primates that are enrolled as part of the National Institutes of Health and National Institute of Allergy and Infectious Diseases-supported Radiation Late Effects Cohort and resource at Wake Forest University School of Medicine^39^. Non-human primate primary neuronal culture was prepared similarly as described in the murine primary neuronal culture following the published study^38^. Briefly, thoracic DRGs of male Rhesus macaques, WFU #1734 (22-year-old) and #1916 (13-year-old), were dissected, directly placed in a 15 mL conical tube containing 14 mL of ice-cold HBSS without Mg++/Ca++ (Fisher Scientific), and transferred to the laboratory for further procedures. Isolated DRGs were cleaned up and were digested through a series of enzymatic treatments using papain and CLS2/Dispase II solutions. Afterwards, single-cell suspended DRG neurons were filtered and further purified by centrifugation in 3.5% (W/V) BSA solution, Cells were seeded onto the center of 12 mm round coverslips, pre-coated with Poly-D-lysine and laminin, in 24-well plate. After 72hrs, cells were treated with radiation using Cs137 γ-rays to a total dose of 10 Gy.

### Culturing and differentiation of osteoclast

The murine pre-osteoclast cell line, Raw264.7 from ATCC was cultured in αMEM media supplemented with 10% FBS, 1% PGS, and 1% glutamine. Cells were passaged every 2–3 days when cells were close to 80% confluent. Cells passaged less than 15 times were used for all the experiments. To differentiate Raw264.7 cells, 3000 cells were plated in 96-well plates or 3.2 ×10^4 cells in 24-well plates in growth media containing 35ng/ml of murine recombinant RANKL (R&D systems, 462-TEC-010). Differentiated osteoclasts were detected at day 3 post addition of RANKL. For co-cultures, 2000 cells/60ul growth media were plated on μ-Slide 2 Well Co-Culture plates (81806, iBidi) in the presence of 35ng/ml RANKL. To inhibit osteoclast differentiation and activity, osteoclast cultures were treated with recombinant murine osteoprotegerin (OPG, R&D systems, 459-MO-100) or Bisphosphonate (Risedronate, Selleckem, S1874) a day before radiation treatment.

### Tartrate-resistant acid phosphatase (TRAP) staining and image analysis

Cells were washed with 1x PBS solution and fixed with a 10% glutaraldehyde solution (Sigma) for 15 minutes at 37°C. The cells were then washed twice with pre-warmed 1x PBS and stained with TRAP staining solution containing 0.3 mg of Fast Red Violet LB Salt per ml of TRAP buffer [50 ml of 0.1 M acetate solution, 10 ml of 0.3 M sodium tartrate, 1 ml of 10 mg/ml Napthol solution, 100 μl of Triton X-100, and 38.9 ml of ddH2O, pH 5] for 10 minutes at 37°C. After removing the TRAP staining solution, the cells were washed with 1x PBS and air-dried. Images were acquired on an Olympus IX70 inverted microscope with a DP80 camera at 10x magnification. Four images were taken for each well. The images were captured and processed with CellSens software (Olympus). Using Nikon image analysis software, TRAP-positive osteoclasts were masked, and the total area masked was calculated and normalized to the total number of osteoclasts within each image to demonstrate changes in osteoclast maturation and activity level.

### Resorption pit assay for osteoclast

Raw264.7 cells (3×103) were seed on each 96-well OsteoAssay plates (Corning) in 100ul of growth media containing RANKL (35ng/ml). 3 days after differentiation, cells were exposed to radiation. 48hr after radiation treatment, cells were removed with 10% bleach solution for 5 min. Washed each well with distilled water. Each well was stained with 1% toluidine blue solution for 10 mins at room temperature, washed with distilled water and air dried. Images of individual pits or multiple pit clusters were acquired on an Olympus IX70 inverted microscope with a DP80 camera at 10x magnification.

### Conditioned media (CM) generation

To collect the CM of radiation activated mature osteoclasts, 3.2 ×10^4 of RAW264.7 cells were seeded on 24-well plates in the presence of 35ng/ml RANKL. After 3 days of differentiation, cells were re-fed with fresh growth media with RANKL (35ng/ml) and treated with 10Gy radiation using Cs137 γ-rays. The control cells were cultured in the same manner but were not treated with radiation. The cells were then maintained in a 37°C incubator. After 48 hrs, media was collected, centrifuged at full speed for 2 min to remove debris, and supernatant was transferred to new tubes.

### RT-qPCR

Cells were lysed in 350 μL of RLT-β-ME buffer. RNA was extracted using the Universal RNeasy plus kit (Qiagen, Germantown, MD) and cDNA was generated using Ezdnase enzyme (11766051, Thermo Fisher Scientific) and SuperScriptTM IV VILO mater mix (11-756-050, Thermo Fisher Scientific) following the company’s protocol. Real time qPCR (RT-qPCR) was performed using TaqMan Universal PCR master mix (Applied Biosystems, Foster City, CA) and TaqMan gene expression assays on the Applied Biosystem PCR instrument. TaqMan gene expression assays used: CGRP/Calca (Mm00801463_g1) and Substance P/Tac1 (Mm01166996_m1) were used for pain associated biomarkers. CTSK (Mm00484039_m1), RANK/Tnfrsf11a (Mm00437132_m1), MMP9 (Mm00442991_m1), DcStamp (Mm04209236_m1) were used for osteoclast cell markers. Data is presented using the delta-delta Ct method, with GAPDH (Mm99999915_g1) or β-actin (Mm02619580_g1) used as the reference gene.

### Immunofluorescence staining

Fixed cells were blocked with immunofluorescence (IF) buffer [1x D-PBS supplemented with 5% Normal Donkey serum (Jackson ImmunoResearch, West Grove, PA) and 0.03% Triton X-100 (Sigma-Aldrich)] for 1h at RT and incubated with primary antibodies overnight at 4°C in IF buffer. Primary antibodies used: mouse anti-β III tubulin antibody (1:1,000, Biolegend, San Diego, CA, cat #: 801201); chicken anti-200kD neurofilament (NF200) antibody (1:3,000, Neuromics, Cambridge, MA, cat #: CH22104); rabbit anti-calcitonin gene-related peptide (CGRP) antibody (1: 5,000, Sigma-Aldrich, cat #: C8198). Afterwards, the cells were incubated with secondary antibodies for 2h at RT in IF buffer. Secondary antibodies used: anti-rabbit cyanine 3 (CY3) (1:700, Jackson ImmunoResearch, cat #: 711-165-152); anti-mouse CY2 (1:600, Jackson ImmunoResearch, cat #: 715-225-150); anti-chicken CY5 (1:500, Jackson ImmunoResearch, cat #: 703-225-155). After washing 5 times with 1x D-PBS, the cells were mounted with ProLong Gold antifade mountant with DAPI (Thermo Fisher Scientific).

### Imaging analysis

For each group, a total of 3 images (1 large image/coverslip for triplicates) were taken using a Nikon Eclipse Ni fluorescent microscope system (Nikon, Tokyo, Japan) to capture the total neuronal culture. Images were saved as nd2 files for further analysis using Visiopharm following the previously published study (Hørsholm, Denmark). An algorithm (called an “APP”) created in the Visiopharm software was modified to mask total soma and neurites of the neurons in images taken for each experiment. Batch analysis was run for each study using the app to generate the total nerve length and soma count.

### Statistical analysis

Numerical data are expressed as mean ± the standard error of the mean (SEM). Statistical analysis was performed by unpaired two-tailed Student’s t test or one-way ANOVA with Tukey’s multiple comparisons, using the GraphPad Prism statistical program (GraphPad Software, San Diego, CA) with significance at p ≤ 0.05.

## Data Availability

The datasets used and/or analyzed during the current study available from the corresponding author on reasonable request
